# Winners always win: growth of a wide range of plant species from low to future high CO_2_

**DOI:** 10.1002/ece3.1687

**Published:** 2015-10-15

**Authors:** Andries A. Temme, Jin Chun Liu, William K. Cornwell, Johannes H. C. Cornelissen, Rien Aerts

**Affiliations:** ^1^ Department of Ecological ScienceVU UniversityDe Boelelaan 10851081HVAmsterdamThe Netherlands; ^2^ Key Laboratory of Eco‐Environment in Three Gorges Reservoir RegionSchool of Life ScienceSouthwest UniversityBeibeiChongqing400715China; ^3^ Ecology and Evolution Research CentreSchool of Biological, Earth, and Environmental SciencesUniversity of New South WalesKensington 2052SydneyNew South WalesAustralia

**Keywords:** Growth response, high CO_2_, low CO_2_, morphology, plant types, relative growth rate, trade‐off

## Abstract

Evolutionary adaptation to variation in resource supply has resulted in plant strategies that are based on trade‐offs in functional traits. Here, we investigate, for the first time across multiple species, whether such trade‐offs are also apparent in growth and morphology responses to past low, current ambient, and future high CO_2_ concentrations. We grew freshly germinated seedlings of up to 28 C_3_ species (16 forbs, 6 woody, and 6 grasses) in climate chambers at 160 ppm, 450 ppm, and 750 ppm CO_2_. We determined biomass, allocation, SLA (specific leaf area), LAR (leaf area ratio), and RGR (relative growth rate), thereby doubling the available data on these plant responses to low CO_2_. High CO_2_ increased RGR by 8%; low CO_2_ decreased RGR by 23%. Fast growers at ambient CO_2_ had the greatest reduction in RGR at low CO_2_ as they lost the benefits of a fast‐growth morphology (decoupling of RGR and LAR [leaf area ratio]). Despite these shifts species ranking on biomass and RGR was unaffected by CO_2_, winners continued to win, regardless of CO_2._ Unlike for other plant resources we found no trade‐offs in morphological and growth responses to CO_2_ variation, changes in morphological traits were unrelated to changes in growth at low or high CO_2_. Thus, changes in physiology may be more important than morphological changes in response to CO_2_ variation.

## Introduction

From slow‐growing cypresses to prolific kudzu vines, plants employ a wide variety of different growth strategies depending on environmental resource availability (Bloom and Mooney 1985). Plant growth not only depends on external resources such as light, carbon dioxide, water, and nutrients but also on plant morphology and photosynthetic capacity and their underlying traits. Due to constraints and trade‐offs in evolution of plant functioning, no single plant species has solutions to cope with more than a limited fraction of the environmental variation in space and time. Trait combinations that result in high growth rates in one environment may preclude good performance in another environment. Such trade‐offs are widespread in the plant kingdom, and for light, nutrients, and water, they have been analyzed in great detail (e.g., Aerts and Chapin 2000; Diaz et al. 2004; Wright et al. 2004; Feschet et al. 2010; Reich 2014). These trade‐offs underpin the current understanding of plant strategy theory (Grime 2006). Given the trade‐offs observed for other growth‐related resources, it seems logical to assume that they must be present for CO_2_ as well, as indicated previously by plant responses to high CO_2_ concentrations as predicted for the latter part of this century (Poorter and Navas 2003). However, such trade‐offs have not been analyzed for a substantial set of species over the whole range from Pleistocene via ambient to future CO_2_ concentrations.

Carbon dioxide is special as it shows little spatial variation. All over the globe, CO_2_ concentrations in open vegetation show only limited variation with season, latitude, and elevation (Peters et al. 2007). However, currently, plants worldwide are faced with rapidly increasing CO_2_ concentrations: CO_2_ will double from the current 400 ppm to 700–800 ppm by the end of this century (Collins et al. 2013). At an evolutionary timescale, the variation in CO_2_ has been even larger, ranging from 3000 ppm in the Devonian down to 180 ppm during the Pleistocene Ice Age (Royer 2006; Hönisch et al. 2009). It is only after the industrial revolution that CO_2_ levels started to rise from 280 ppm to the 400 ppm we have today. Thus, compared to the current situation, recent plant evolution has been at a low level of atmospheric CO_2_.

Plant responses to environmental factors are often treated categorically using the PFT (plant functional type) concept (Chapin et al. 1996), which groups plant species by their similar adaptations and responses to certain environmental factors. As a result of different growth form strategies and physiological mechanisms, plants could show contrasting responses to shifts in CO_2_ concentration. For example, woody plant species invest a lot of their biomass in nonphotosynthetic stem tissue (Poorter et al. 2012b), so in early stages of development at low CO_2,_ they might be outcompeted by grasses or forbs that can invest more in carbon acquiring leaf tissue (Bond and Midgley 2012). However, at high CO_2_, their observed greater biomass accumulation in perennial tissues (Ainsworth and Long 2005) might lead them to outcompete grasses and forbs. Differences in direction and magnitude of trait responses to CO_2_ between plant functional types could thus lead to shifts in competitive interactions.

In terms of carbon capture, the RGR (relative growth rate, g g^−1^ day^−1^) of plants depends on two aspects: leafiness and physiology. This is encapsulated in the equation RGR = LAR*ULR (Evans 1972), in which RGR is dependent on LAR (leaf area ratio, m^2^ leaf per g plant) and ULR (unit leaf rate, g plant grown per m^2^ leaf per day). Differences in carbon capture may be driven by either: the chemical and physiological traits underlying ULR; tissue carbon content and photosynthesis rate; or the allocation and morphology traits underlying LAR, leaf mass fraction (LMF, leaf mass per unit plant mass), and SLA (specific leaf area, leaf area per unit dry mass) (Lambers and Poorter 1992). At low CO_2_, a high SLA might be advantageous because of reduced diffusive resistance in the leaf (Loreto et al. 1992; Medlyn et al. 2011) and serve to increase the area available for photosynthesis at a lower carbon cost to biomass. A higher biomass allocation to leaves (LMF) serves to take up more of the most limiting resource, carbon, required for optimal growth (Bloom and Mooney 1985). At high CO_2_, these traits show less return upon investment due to increased CO_2_ availability. With an abundant availability of carbon other factors determining growth such as nutrient uptake rate and light availability can become more limiting (Poorter and Pérez‐Soba 2001; Lewis et al. 2010). Physiologically, at low CO_2_, photosynthetic rates are limited by RuBisCO carboxylation rate and thus more nitrogen invested in the photosynthetic machinery would increase carbon gain per unit of time (Sage and Coleman 2001; Ripley et al. 2013). Again, at high CO_2_ that high nitrogen investment is less beneficial and nitrogen could be used elsewhere, for instance to speed up RuBP regeneration (Makino et al. 2000). Trade‐offs in plant design thus lead to different patterns of carbon capture and processing at low versus high CO_2_. But how do different plant species and PFTs vary in their traits and associated growth performance across the whole range from low to high CO_2_ while obeying such tradeoffs?

While there is ample data on plant species response to elevated CO_2_ (Poorter and Navas 2003; Ainsworth and Rogers 2007; Norby and Zak 2011), far less is known on plant responses to low CO_2_ (reviewed in Gerhart and Ward 2010; Temme et al. 2013). A previous analysis of literature data revealed that the response of plant species to low and high CO_2_ is opposite both in magnitude and direction and that plant trait adjustments to low CO_2_ are far greater than to high CO_2_ (Temme et al. 2013). With consequently greater effects on C and N cycling (Gill et al. 2002) at low CO_2_. At high CO_2_, only moderate increases in biomass are found (perhaps due to the saturating nature of CO_2_ capture) with a small decrease of SLA and LAR (Poorter and Navas 2003; Ainsworth and Rogers 2007; Norby and Zak 2011). Thus, at increased CO_2_ concentrations, plant morphological and growth responses (lower SLA, higher RGR) move away from the trait values that, at the interspecific levels, are associated with fast growth (higher SLA = higher RGR), possibly due to a disproportionate increase in photosynthesis per unit leaf area. The very limited experimental results so far have shown that at low CO_2_ representing the Pleistocene Ice ages a whole suite of traits is drastically altered compared to ambient CO_2_. Morphological traits are strongly adjusted in response to low CO_2_. Thinner and less dense leaves (Smith et al. 2012) lead to a much higher SLA. Combined with an increase in LMF, this results in a higher LAR (Gerhart and Ward 2010; Temme et al. 2013). Plant morphological traits at low CO_2_ are thus adjusted toward the trait spectrum of today's fast growers. However, despite these substantial phenotypic responses, resource starvation is such that there is nevertheless a strong reduction in biomass, amounting to up to 90% for some species (Temme et al. 2013). Trait shifts thus may ameliorate some of the effects of low CO_2_ but are insufficient to entirely compensate for the diminished concentration of the resource.

Current knowledge makes it difficult to determine how the relationships between leaf morphology, plant allocation and growth rate have changed from past to present atmospheric CO_2_ concentrations, and how these relationships compare to the responses of today's plant species to future CO_2_ concentrations. Thus, our study strives for generality and addresses responses of morphological and allocation traits and their links to growth performance from low to high atmospheric CO_2_ concentrations for a wide range of species in the same experiment. It will also shed light on the question whether, among diverse species, the winners in terms of growth performance at current CO_2_ would still be the winners at low or high CO_2_.

Thus, our study had the following research questions: (1) Do species‐specific responses in RGR at low and high CO_2_ as compared to ambient CO_2_ affect the ranking of species in RGR? To put it differently: Are the winners in today's atmosphere also the winners at low and at high atmospheric CO_2_? (2) Which plant functional types (woody, grass, forb) lose or will gain the most in terms of growth rate at low and high CO_2,_ respectively? (3) How are changes in RGR related to changes in underlying allocation and morphological traits?

To that end, we performed an experiment to quantify variation in growth rate and morphological and allocation traits among 28 different species belonging to a wide variety of C_3_ plant functional types in walk‐in climate chambers at a wide range of CO_2_ concentrations, 160 ppm, 450 ppm, and 750 ppm CO_2_.

## Materials and Methods

### Species

To determine the response of a variety of plant growth forms to variation in CO_2_ concentration, we obtained seeds from a wide range of temperate (and partly subtropical) woody, forb, and grass C_3_ species. These had been field collected in Sheffield, U.K., and the Chongqing region, SW China, as well as supplied by B&T World Seeds, Sheffield Seed co., USA, and Kruythoeck Seeds, Netherlands. The seeds were set out to germinate by placing them on either wet sand or wet tissue paper. Special pretreatment of seeds (scarification, soaking, hot/cold shock) was carried out according to supplier instructions and the authors' experience. This resulted in successful germination of 28 different species (Table S1) which consisted of six woody species (2 trees, 4 shrubs), 16 forb species, and 6 grasses. Shortly after germination, individual plants were transferred to experimental CO_2_ conditions at the Phytotron labs at Utrecht University, The Netherlands. The growth experiment was spread out over the period October 2012 – October 2013, during which batches of different species were sequentially screened in the standardized environmental regimes.

### Growth conditions

We used three separate custom‐built walk‐in climate rooms (Reftech B.V., Sassenheim) in which we maintained three CO_2_ levels: low, ambient, and high. These levels broadly (±50 ppm) represented the large range from Pleistocene past to future high CO_2_. The low CO_2_ concentration of 160 ppm (peaking to 180 ppm when handling the plants inside the chamber) was achieved by scrubbing CO_2_ from ambient air ventilating the room using a molecular sieve (PG 1500L, CMC Instruments GmbH, Eschborn). The ambient level (450 ppm) was slightly higher than outside air due to elevated levels inside the office building. The high level (750 ppm) was achieved by adding fossil fuel derived CO_2_ from high‐pressure tanks to ambient air in the climate room. CO_2_ levels inside the chambers were digitally monitored (GMP343, Vaisala GmbH, Bonn) and scrubber or valve capacity adjusted accordingly. Low levels of CO_2_ while handling plants were maintained using a gas mask to capture exhaled breath in a large airtight bag.

Growth conditions were ~350 *μ*mol light, 18°C night/21°C day temperature, 10‐h photoperiod, and 70% relative air humidity. Total daily photon flux was comparable to that of an average March day in the Netherlands, which is when several of the species would have naturally germinated and start to grow. Pots were watered thrice daily up to field capacity using an automated watering system supplying water from below. To prevent nutrient limitation during the experiment, nutrients were added three times per week with 50 mL full Hoagland solution (6 mmol L^−1^ KNO_3_, 4 mmol L^−1^ Ca(NO_3_), 2 mmol L^−1^ NH_4_H_2_PO_4_, 1 *μ*m KCl, 25 *μ*m H_3_BO_3_, 2 *μ*m MnSO_4_, 2 *μ*m ZnSO_4_, 0.1 *μ*m CuSO_4_, 0.1 *μ*m (NH_4_)_6_Mo_7_O_24_, 20 *μ*m Fe(Na)EDTA). To prevent damage from excess nutrients to young plants, freshly germinated individuals were supplied with an increasing concentration starting with 25% nutrients after germination to full Hoagland at the onset of the first leaf and subsequent growth period.

Shortly after seed burst and germination at ambient CO_2_, seedlings were transferred to the CO_2_ chambers in 400‐mL plastic pots containing coarse sand. Because of the small size, the seedlings were expected to obtain during the duration of the experiment pot size was assumed to be sufficient to maintain less than 2 g plant per L soil to avoid pot size effects (Poorter et al. 2012a). Although we did not observe any strong symptoms of pot boundness, we cannot entirely exclude such effect in the largest plants (see Discussion). We tried to standardize the period of the exponential growth phase based on the ontogenetic phase of the plants at the start of this period. After expansion of the first leaf (as in Cornelissen et al. 1996), a representative subset of each species (4–8 individuals depending on germination success) was harvested and oven dried at 70°C for 48 h as a baseline biomass measure of the total set of individuals. Subsequently, plants were grown for three more weeks after which the remaining ±7 individuals (Table S1) were harvested. Due to the small plant size and young age, growth during these 3 weeks was assumed to be in the exponential phase (Grime and Hunt 1975). Using the baseline biomass and final biomass, we could calculate RGR (Hoffmann and Poorter 2002). Because of space constraints, species were staggered in six batches of species where the experimental regime in the climate chambers was held constant and continuously monitored.

### Final harvest

**Figure 1:**
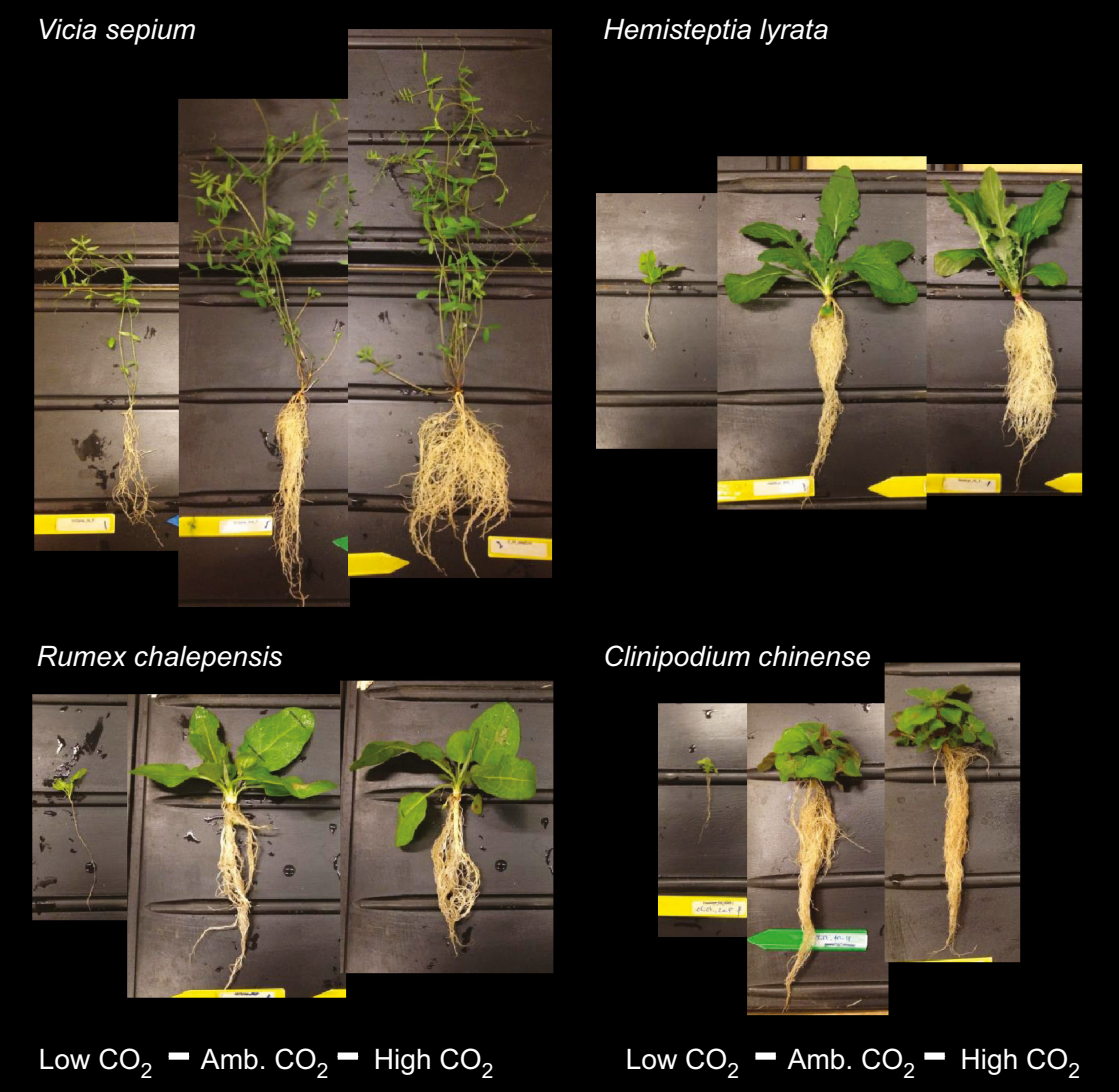
Plants grown at low, ambient, and high CO_2_. Images illustrate the response of four plant species, *Vicia sepium, Hemisteptia lyrata, Rumex chalepensis,* and *Clinopodium chinense,* to growth at 1 ppm, 450 ppm, 750 ppm CO_2_.

At final harvest, plants were washed to remove sand from roots, and fresh weight (weighed to the nearest mg) was measured for above and belowground plant parts. Images were taken to illustrate the effects of CO_2_ on plant size (Fig. 1). Leaf area was measured by scanning (Canon LiDe 110 at 300dpi) a representative full‐grown leaf for SLA (m^2^_leaf_ g^−1^_leaf_ dry weight) measurements. Leaf area (m^2^) was then determined by pixel counting using ImageJ version 1.47. Fresh plant material was oven dried at 70°C for 48 h and weighed. After drying, leaves were removed from stems and stems were weighed to calculate leaf and stem mass fraction. Plant leaf area ratio (LAR, m^2^_leaf_ g^−1^_plant_) was then calculated by multiplying SLA with leaf mass fraction.

### Statistics

Due to difficulties in germinating enough seedlings to do a representative baseline harvest, RGR of three species *(Buddleja davidii, Clinopodium chinense, Stellaria media)* could not be determined. Two episodes where the low CO_2_ and high CO_2_ chamber were unavailable due to emergency maintenance led to 25 species in the low CO_2_ treatment, 22 species in the high CO_2_ treatment and 19 species with all three treatments.

Given the limited number of species analyzed for low to high CO_2_ response (Gerhart and Ward 2010; Temme et al. 2013), we felt that a higher number of species would further our understanding of plant responses to CO_2_ more than an in depth look at a limited number of species with more chamber replicates. As this entails some danger of pseudoreplication, we tested the robustness of our approach by (1) measuring a single species at multiple time intervals; and (2) comparing the results of multiple batches of different species at different times. Data on RGR and SLA for *Avena sativa* grown in different batches with substantial time intervals showed a consistent response to CO_2_ and supports the robustness of the treatment in the climate chambers (Figure S1); only one batch was used in this study. While different species grown during different batches show moderately different responses to CO_2_, the overall effect of low or high CO_2_ was comparable between species and batches (Figure S2). We are thus confident that a repeat of the experiment with a different “random draw” of species would lead to similar conclusions.

Results were analyzed using R (version 3.0.1, R Core Team, Vienna, Austria) and RStudio (version 0.98, RStudio, Inc., Boston, MA). Changes in the interspecific ranking based on RGR were analyzed nonparametrically by determining species rank on RGR at low, ambient, and high CO_2_. Rank changes were then tested pairwise between CO_2_ treatments in a paired‐Wilcoxon‐signed‐rank test with Bonferroni correction. CO_2_ effects on traits and species differences in traits were tested by comparing the shift in trait value to the trait value at ambient CO_2_. To improve normality and minimize skew, trait values were natural log (ln) transformed prior to analyzing CO_2_ effect on trait shifts. The difference in ln‐transformed trait level from ln‐transformed ambient level was then the relative shift in trait level via e^ln transformed difference^−1. This approach had the added benefit that a halving or a doubling in trait value from ambient had the same ln‐transformed difference. Per species we averaged the trait response of individual replicates per treatment. These shifts in species trait values at low or high CO_2_ (compared to ambient CO_2_) were then tested by one‐sample *t*‐tests. Differences between plant types were determined by two‐sample *t*‐tests on species trait shift with Bonferroni correction for the three comparisons made (forb‐grass, forb‐woody, grass‐woody). To determine whether the reduction and stimulation in growth and biomass was related to trait values at ambient CO_2_ or shifts in trait value toward low or high CO_2_, we performed a stepwise model selection procedure selecting models based on AIC using the MASS package (version 7.3, Venables and Ripley 2002). The initial model to determine whether trait values at ambient CO_2_ were related to RGR and biomass differences included RGR or biomass at ambient CO_2_ and RMF (root mass fraction), LMF (leaf mass fraction), and LAR at ambient CO_2_. The initial model to test the relationship between the difference in RGR and shifts in trait level included the shifts in RMF, LMF, and LAR. The relationship between RGR and LAR at all three CO_2_ levels was determined using ordinary least squares regression as we viewed LAR as a predictor of RGR.

## Results

Plants responded strongly to the low and high CO_2_ treatments. The photographs of Figure 1 illustrate the large effect of CO_2_ on plant size. In general, plants at low CO_2_ were tiny compared to ambient CO_2_ and as expected plants were stimulated by elevated CO_2_. While different species showed moderately different responses, species grown in different batches in different periods showed comparable responses to CO_2_ (Figure S2).

### Species ranking on RGR and biomass

**Figure 2:**
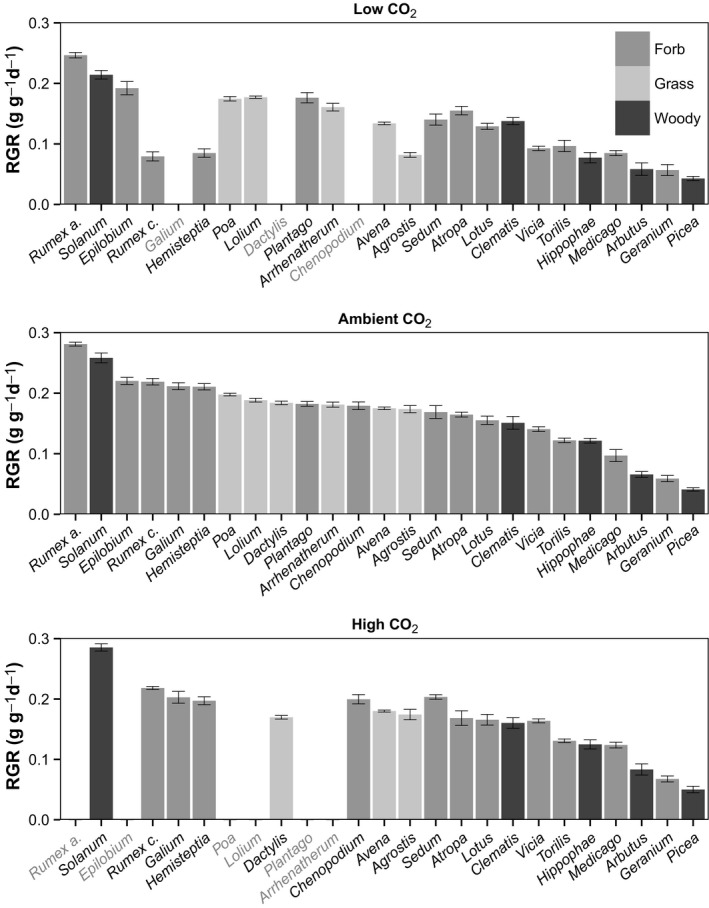
Plant species RGR (relative growth rate, g g^−1^ day^−1^) ranking at 160 ppm, 450 ppm and 750 ppm CO_2_. Species are ordered by RGR at 450 ppm CO_2_. Light gray species names indicate species is missing at this CO_2_ treatment. Light‐gray bars: grass species, medium‐gray bars: forb species, dark‐gray bars: woody species. Error bars denote SE.

**Figure 3:**
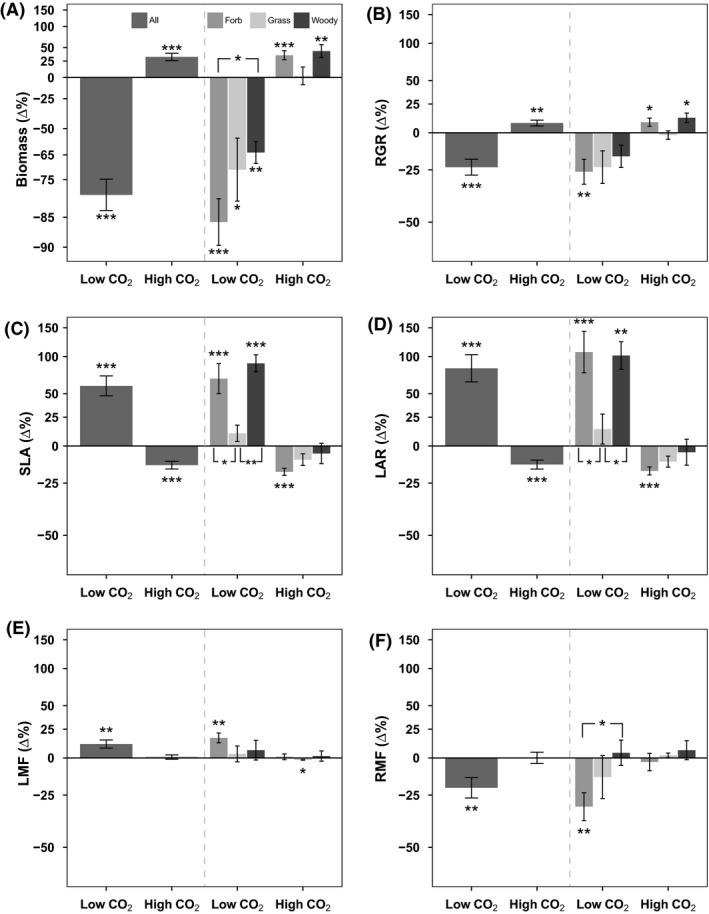
Relative shift in trait level at low or high CO_2_ compared to ambient CO_2_ for forb, grass, and woody species. Bars indicate percentage shift in trait value at low CO_2_ (160 ppm) and high (750 ppm) CO_2_ compared to trait value at ambient (450 ppm) CO_2_. Axes are natural log transformed so that the size of the bars at a 50% decrease or a 100% increase is the same (reflecting a factor 2 adjustment). Left section: all species (25 low/22 high), right section: medium‐gray bars: forb species (14 low/13 high), light‐gray bars: grass species (5 low/3 high), dark‐gray bars: woody species (6 low/6 high). Error bars give SE. *s near error bars indicate *t*‐test significance from zero. *s opposite bars indicate significance of 2‐sample *t*‐test between linked types. *: *P* < 0.05, **: *P* < 0.01, ***: *P* < 0.001. (A) Biomass, dry weight of plants (g). (B) RGR (Relative growth rate, g g^−1^ day^−1^), two less forbs and one less woody species. (C) SLA (Specific leaf area, m^2^_leaf_  g^−1^_leaf_). (D) LAR (Leaf area ratio, m^2^_leaf_  g^−1^_plant_). (E) LMF (Leaf mass fraction), g_leaf_  g^−1^_plant_. (F) RMF (Root mass fraction, g_root_ g^−1^_plant_).

Species varied over 6‐fold in their RGRs with the woody gymnosperm *Picea sitchensis* growing the slowest regardless of CO_2_ concentration and the forb *Rumex acetosella* (missing at high CO_2_) and semi‐woody scrambler *Solanum dulcamara* growing fastest at low, ambient, and high CO_2_ (Fig. 2). Figure 2 shows that at low and high CO_2_, there were only minor shifts in the ranking of species on RGRs as compared to the ranking at ambient CO_2_: Fast growers tended to grow relatively fast and slow growers grew relatively slowly irrespective of CO_2_ treatment. This was confirmed by pairwise‐Wilcoxon‐signed‐rank tests which showed no significant changes in species ranking on RGR between low, ambient, and high CO_2_. Averaged over all three PFTs, RGR was reduced by 23.4% ± 4.7 (*P* < 0.001) at low CO_2_ (Fig. 3B). However, likely due to large variation among species and small sample size, the separate response for grass and woody species was not significant. At high CO_2_, RGR increased on average by 7.8% ± 2.5 (*P* < 0.01) although grass species did not increase their RGR (Fig. 3B).

As with RGR, there were only minor shifts in the ranking of plant biomass at the end of the experimental period (Figure S3). Low CO_2_ reduced biomass on average by 79.7% ± 4.3 (*P* < 0.001) and high CO_2_ increased biomass by 32.2% ± 6.7 (*P* < 0.001) (Fig. 3A) but again there were no significant changes in species ranking.

**Figure 4:**
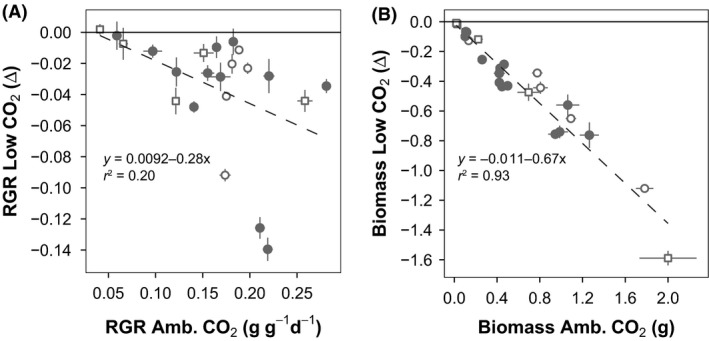
Difference in growth rate and plant biomass at past low (160 ppm) CO_2_ compared to current ambient (450 ppm) CO_2_. (A) Relative growth rate difference at low CO_2_ shows a negative relationship (black line) to growth rate at ambient CO_2_, species that grow fast at 450 ppm CO_2_ are more reduced in growth rate than slow‐growing species. *R* ^2^ = 0.20, *P* < 0.05. (B) Biomass difference at low CO_2_ shows a negative relationship with biomass at ambient CO_2_ (black line), that is, bigger species lose more biomass at low CO_2_. *R* ^2^ = 0.93, *P* < 0.001. Closed circles: forb species, open circles: grass species, open squares: woody species. Error bars give SE.

While the ranking of species for RGR and biomass was not significantly altered by CO_2_, we did find that, in general, species with a higher RGR at ambient CO_2_ had a stronger reduction in RGR with low CO_2_ (Fig. 4A). As the ranking of species on RGR remained similar across CO_2_ levels and fast growers at ambient were more affected by low CO_2_, this had the effect that in the community of species the difference in RGR between the top‐ranked and bottom‐ranked species was reduced at low CO_2_ (Figure S4). These RGR differences led to absolute biomass loss at low CO_2_ compared to ambient being highest for large species (Fig. 4B) while relative biomass loss was overall not significantly different between species, although the largest woody species had a greater reduction then the smaller species (Figure S5). No such results were found at high CO_2_ however (Figure S6).

### Differences between plant types

Plant types showed only limited differences in their responses to CO_2,_ only at the stress of low CO_2_ did we find significant differences in trait adjustment between types (Fig. 3). Biomass loss was different between forbs and woody species with forbs having a 85.9% ± 4.4 reduction in biomass and woody species only a 63.9% ± 5.5 reduction. Between woody and forb species, the adjustment in RMF (root mass fraction) was significantly different as well (Fig. 3F) with woody species not adjusting RMF but forbs decreasing RMF by 16.9% ± 4.4 (*P* < 0.01). Grass species had a markedly different response in SLA and LAR. On average, relative to ambient CO_2_, SLA increased strongly at low CO_2_ by 59.4% ± 12.4 (*P* < 0.001) and decreased modestly by 13.8% ± 2.6 at high CO_2_ (*P* < 0.001). However, when viewed separately at low CO_2_, woody and forb species had a very large increase in SLA (68.6% and 89.8%, respectively) whereas grass species did not significantly increase their SLA (Fig. 3C). At high CO_2_, forb species decreased SLA by 18.2% ± 2.3 (*P* < 0.001), and grass and woody species, however, did not significantly reduce SLA. For LAR, a similar result was found (Fig. 3D) with grass species not significantly increasing LAR but a large increase in LAR for forbs (107.1% ± 33.3) and woody species (101.7 ± 21.6) was found at low CO_2_. At high CO_2_, only forbs showed a significant decrease in LAR (17.6% ± 2.6).

### Morphology and growth

**Figure 5:**
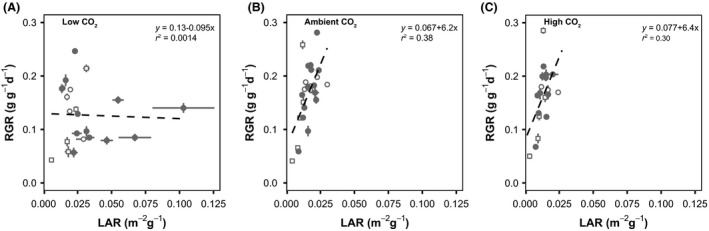
Relationship between LAR (leaf area ratio) and RGR (relative growth rate) at past low (160 ppm), current ambient (450 ppm), and future high (750 ppm) CO_2_. The relationship between LAR and RGR is positive at ambient (B) and high (C) CO_2_ (*P* < 0.05, *R* ^2^ = 0.38 & *R* ^2^ = 0.3, respectively). At low CO_2_, (A) no significant relation is found. Points indicate species mean RGR and LAR with SE; closed circles: forb species, open circles: grass species, open squares: woody species.

In general, plant morphological traits were poor predictors of growth response to CO_2_. None of the morphological traits (RMF, LMF, LAR) was significantly related to differences in growth rate or biomass at low CO_2_. However, species with the highest growth rate or largest biomass at ambient CO_2_ did show the strongest absolute reduction at low CO_2_ (Fig. 4). Shifts in trait value of RMF, LMF, and LAR were also not significantly related to differences in RGR or biomass at low CO_2_. Similarly for high CO_2_, none of the trait shifts or trait values at ambient CO_2_ was significantly related to stimulation of growth or biomass at high CO_2_. This is possibly due to a changed relationship between LAR and RGR at low CO_2_ (Fig. 5). At ambient CO_2_ and high CO_2,_ there was a positive relationship between LAR and RGR (*r* ^2^ = 0.38 & *r* ^2^ = 0.30 respectively, *P* < 0.01). However, at low CO_2_ (Fig. 5A), RGR decreased despite a strong increase in LAR, thus decoupling the generally observed positive relation between RGR and LAR. The LAR–RGR relationships were determined more strongly by SLA than by LMF (Figure S7).

## Discussion

This study is novel in that we investigated 19 plant species belonging to different functional types in their performance across the whole range of Pleistocene low, via ambient to future high CO_2_ levels, and 25 species for their performance at low CO_2_, thereby doubling the available data on plants' low CO_2_ response (Temme et al. 2013). This approach has enabled us to make an experimental analysis of growth responses of plants to variation in CO_2_, and how these are related to changes in morphological traits. We found that, while RGR and plant biomass were strongly affected by both low and high CO_2_, the ranking of species for RGR and biomass was not affected. Thus, we did not find a classical “trade‐off” by which species with faster growth in response to low CO_2_ compared to other species would have grown relatively slowly at high CO_2_, and *vice versa*. This could be because while CO_2_ concentration can act as a selective force (Ward et al. 2000; Mohan et al. 2004), in open vegetation, there is little spatial variation in CO_2_. As such, unlike for all other plant resources (see Aerts and Chapin 2000), there cannot be selection at any given time for high and low CO_2_ specialists. The morphological explanation for this lack of trade‐off might be that, contrary to what we expected, changes in RGR were unrelated to changes in leaf morphology (SLA) and allocation (LMF, RMF), and thereby to changes in leaf area ratio. Indeed, the well‐established positive relationship between LAR and RGR, as seen at ambient and high CO_2_, broke down entirely at low CO_2_.

### Species rankings on RGR unaffected by CO_2_

In general in plant strategy theory, there are trade‐offs in species performance across resource supply gradients. For example, species that perform well at high nitrogen supply are poor performers at N‐limited growth conditions (Aerts and Chapin 2000) and we expected a similar pattern for CO_2_. However, with some exceptions, species ranking remained similar (Fig. 2). These exceptions are *Rumex chalepensis, Hemisteptia lyrata* and *Agrostis capillaris* which dropped considerably in RGR ranking at low CO_2_. Still, the fast growers at ambient CO_2_ are generally also the fast growers at low and high CO_2_ (Fig. 2). Although the ranking on growth remained the same, it is the fast growers at ambient CO_2_ that suffer from a stronger reduction in RGR at low CO_2_. In terms of *absolute* biomass loss, this pattern is even clearer as there is a strong connection between plant biomass at ambient CO_2_ and biomass reduction at low CO_2_ (Fig. 4). Interestingly, the *relative* biomass loss is not significantly related to plant biomass. Larger plants in general have a similar percentage of biomass reduction at low CO_2_ as smaller plants (Figure S5). This poses the question whether there are clearer winners and losers in interspecific competition now than in the Pleistocene past due to increased differences in growth rate between species.

The interactive effect of CO_2_ with other resources and environmental factors does, however, modulate different plants species response to CO_2_. Limiting N and P supply changes plants response to increasing CO_2_ (Grunzweig and Korner 2003; Lewis et al. 2010; Ripley et al. 2013) as does water supply (Ward et al. 1999; Medeiros and Ward 2013) and temperature (Cowling and Sage 1998; Ward et al. 2008). Indeed, species response to environmental change since the Last Glacial Maximum (LGM) had a greater effect on conifer stand community composition then species response to CO_2_ increase (Becklin et al. 2014). This shows that while our results provide potential shifts in species relative competitive ability due to CO_2_, understanding how changes in resources and the environment interact with CO_2_ is important for understanding shifts in community composition since the LGM. Furthermore in dense canopies, a vertical gradient in CO_2_ can occur with elevated CO_2_ close to the soil (Medina et al. 1986) and depleted (down to 280–300) in the canopy during peak photosynthesis times (Bazzaz and Williams 1991). It could be that for species that occur only in those zones, there might be selection for high and low CO_2_ specialists.

Based on the literature, we expected that, at high CO_2_, fast‐growing species would be stimulated more than slow growers (Poorter and Navas 2003). However, we found only minor stimulation of RGR and biomass, and this was not related to growth rate or plant biomass at ambient CO_2_. While strong pot boundness was not observed visually, we cannot exclude the possibility of pot size having played a small role in this. Large plants at ambient CO_2_ were at or above the recommended limit of 2 g L^−1^, implying that pot size might have limited growth increase of the largest species at high CO_2_ (Poorter et al. 2012a); see also Figs. 2, S6) although there is evidence of pot size not playing a large role in plants high CO_2_ response (Kerstiens and Hawes 1994). Alternatively while at lower light levels the morphological traits assessed here better explain interspecific variation in plant performance (Evans and Poorter 2001), the relatively low light levels could be a factor in the limited growth response. In natural understory stands, shade‐tolerant species were most stimulated by elevated CO_2_ at low light conditions whereas less shade‐tolerant species showed no stimulation (Hattenschwiler and Korner 2000). Indeed, the six heaviest species at ambient CO_2_ that showed little biomass stimulation (Figure S3) are not generally found in shady habitats. From a resource economics perspective, the extent of CO_2_ stimulation should be dependent on the availability of other resources (Bloom and Mooney 1985). However, the interaction with light has generally been found to be small (Poorter and Pérez‐Soba 2001).

### No major differences in CO_2_ response among plant functional types

We found comparable trait responses in the three plant types. Over the whole range of CO_2_ treatments, only the response of SLA, LAR, and root mass fraction was significantly different between plant types. Forbs and woody species greatly increased SLA at low CO_2_, possibly to reduce mesophyll resistance in the leaf (Loreto et al. 1992; Medlyn et al. 2011) or to produce more carbon acquiring leaf area at a lower biomass expense. Grass species, in contrast, showed no significant increase in SLA at low CO_2_, but this did not lead to a greater reduction in biomass at low CO_2_. Whether this means that grasses are less plastic in their SLA response and maintain growth rates through a different (for instance physiological) mechanism is unclear.

While not significantly different over the whole range of CO_2_, at elevated CO_2_ growth and biomass stimulation was greatest for woody species (cf. Curtis and Wang 1998), for which woody tissues may act as a powerful carbon sink reducing build‐up of photosynthates and slow‐down in photosynthetic rates in the leaves. In contrast, grasses, which aboveground consist mostly of foliage, showed little to no stimulation. The greater stimulation of woody species as compared to grasses at high CO_2_ suggests important ecological implications where seedlings of both types compete, for example, after gap formation in a forest (Loik and Holl 2001) or after savannah fires (Kgope et al. 2010; Bond and Midgley 2012).

### Morphological traits and trait plasticity are poor predictors of CO_2_ response

While all species were reduced in their growth rate, some were more affected than others by low CO_2_, and while the difference was smaller at high CO_2_, there was variation in stimulation there as well (Fig. 2). Through stepwise regression, we sought to identify the source of this variation. We found that morphological traits and shifts in them were poor predictors of shifts in RGR from ambient to low or high CO_2_. Allocation patterns to leaves and roots and leaf area ratio were not related to shifts in growth rate. Species that grew faster at ambient and high CO_2_ were more affected by low CO_2_. From the relationship between LAR and RGR (Fig. 5), it can be seen that while a fast‐growth morphology (high LAR) is related to fast growth at ambient and high CO_2_, surprisingly, there was a decoupling of RGR and LAR at low CO_2_. On average, plant species greatly increased SLA at low CO_2_, a trait generally associated with higher RGR (Poorter and Garnier 2007). However, this seems to have been insufficient to ameliorate the carbon starvation experienced at low CO_2_.

This decoupling or RGR and LAR at low CO_2_ seems to suggest that unit leaf rate (ULR, see Introduction) and underlying plant physiological traits are of greater importance in driving differences in growth rate at low CO_2_. At low CO_2_, plants appear to lose the benefits of a fast‐growth morphology which explains why fast growers are most affected by low CO_2_. Both from paleo‐data and from growth chamber studies we know that nitrogen content and photosynthetic rate are strongly affected by low CO_2_ (Gerhart and Ward 2010; Temme et al. 2013; Becklin et al. 2014). Potentially, plants' capacity to adjust these physiological traits might better explain differences in RGR and biomass at low CO_2_.

### From the past to the present

Plant species have not experienced the low CO_2_ concentrations that occurred during the last glacial maximum for at least 17 Ka (Hönisch et al. 2009) but will likely experience a doubling of CO_2_ in the next 80 years. This is a short period for evolutionary change especially given the rapid rise from 280 ppm to current ~400 ppm CO_2_ since the start of the industrial revolution. RuBisCO as the key enzyme in carbon uptake seems to be fine‐tuned to 200 ppm CO_2_ (Zhu et al. 2004). While there is evidence that CO_2_ can act as a strong selective agent in *Arabidopsis thaliana* (Ward et al. 2000) and *Acer rubrum* (Mohan et al. 2004) at low CO_2_, it remains unclear how much plants have adapted to the higher CO_2_ concentration of today through evolutionary changes or whether they are currently adjusting through plasticity in trait responses.

While perhaps plant species trait levels were different during low CO_2_ episodes in the Pleistocene, we believe the direction and magnitude of change of current plants grown at low CO_2_ to be representative of trait levels during the Pleistocene. Although it should be noted that different families do show different levels of response to global change since the LGM (Becklin et al. 2014). The potential ecological and environmental implications for plant growth and development during glacial times are interesting. The reduced belowground biomass due to a combination of slower plant growth and lower allocation to roots has impacted chemical weathering rates of soil during low CO_2_ periods during the Pleistocene (Beerling et al. 2012). Reduced growth rates with thin, high SLA leaves will have made plants more susceptible to damage from herbivory and made the leaves more palatable to herbivores (Pérez‐Harguindeguy et al. 2003; Poorter et al. 2009), the reduction of which is linked to the extinction of the Pleistocene megafauna (Cowling 2001). Slow growth at low CO_2_ was likely a limiting factor for the origin of agriculture as well (Sage 1995; Cunniff et al. 2008).

Plant growth and development are strongly affected by CO_2_ concentration. Differences in traits between plants grown at today's CO_2_ concentration and past Pleistocene low CO_2_ were far greater than differences in traits between plants grown in today's atmosphere and future high CO_2_ atmosphere. Plant growth at past low CO_2_ concentration was strongly reduced with fast‐growing species being more affected by carbon starvation than slow‐growing species. This had the effect of diminishing RGR differences between fast and slow growers while the ranking of species for growth rate remained broadly similar. Moreover, the greater reduction in growth rate and biomass of fast‐growing species at low CO_2_ is likely associated with the decoupling of more ‘leafy’ (higher SLA, higher LAR) morphology with faster growth. Differences in growth rate at carbon starvation could therefore be driven more by physiological differences. Understanding how physiological traits are affected by carbon starvation and carbon excess will shed more light on the interaction between morphology, physiology, and growth from past low to future high CO_2_.

## Conflict of Interest

None declared.

## Supporting information


**Figure S1** Response of multiple batches of *Avena sativa* to CO_2_.
**Figure S2** Comparison of RGR and SLA response to CO_2_ of two batches of different species.
**Figure S3** Plant species biomass ranking at low, ambient and high CO_2,_
**Figure S4** RGR and RGR rank at low, ambient and high CO_2._
**Figure S5** Relative biomass lost at low CO_2_ as compared to ambient CO_2._
**Figure S6** Difference in growth rate and biomass at high CO_2_ as compared to ambient CO_2_.
**Figure S7** Relationship between leaf mass fraction (LMF) and relative growth rate (RGR) at past low, ambient future high CO_2_.

**Table S1** Species grown and their classification.
