# Supplementary material for: Winners always win: growth of a wide range of plant species from low to future high CO 2
Source: Ecol Evol. 2015 Oct 15;5(21):4949–61. doi: 10.1002/ece3.1687 (PMC4662314; doi:10.1002/ece3.1687)
Supplement: Supplementary file 1 — Figure S1 Response of multiple batches of Avena sativa to CO2. Figure S2 Comparison of RGR and SLA response to CO2 of two batches of different species. Figure S3 Plant species biomass ranking at low, ambient and high CO2, Figure S4 RGR and RGR rank at low, ambient and high CO2. Figure S5 Relative biomass lost at low CO2 as compared to ambient CO2. Figure S6 Difference in growth rate and biomass at high CO2 as compared to ambient CO2. Figure S7 Relationship between leaf mass fraction (LMF) and relative growth rate (RGR) at past low, ambient future high CO2. [file ECE3-5-4949-s001.pdf]

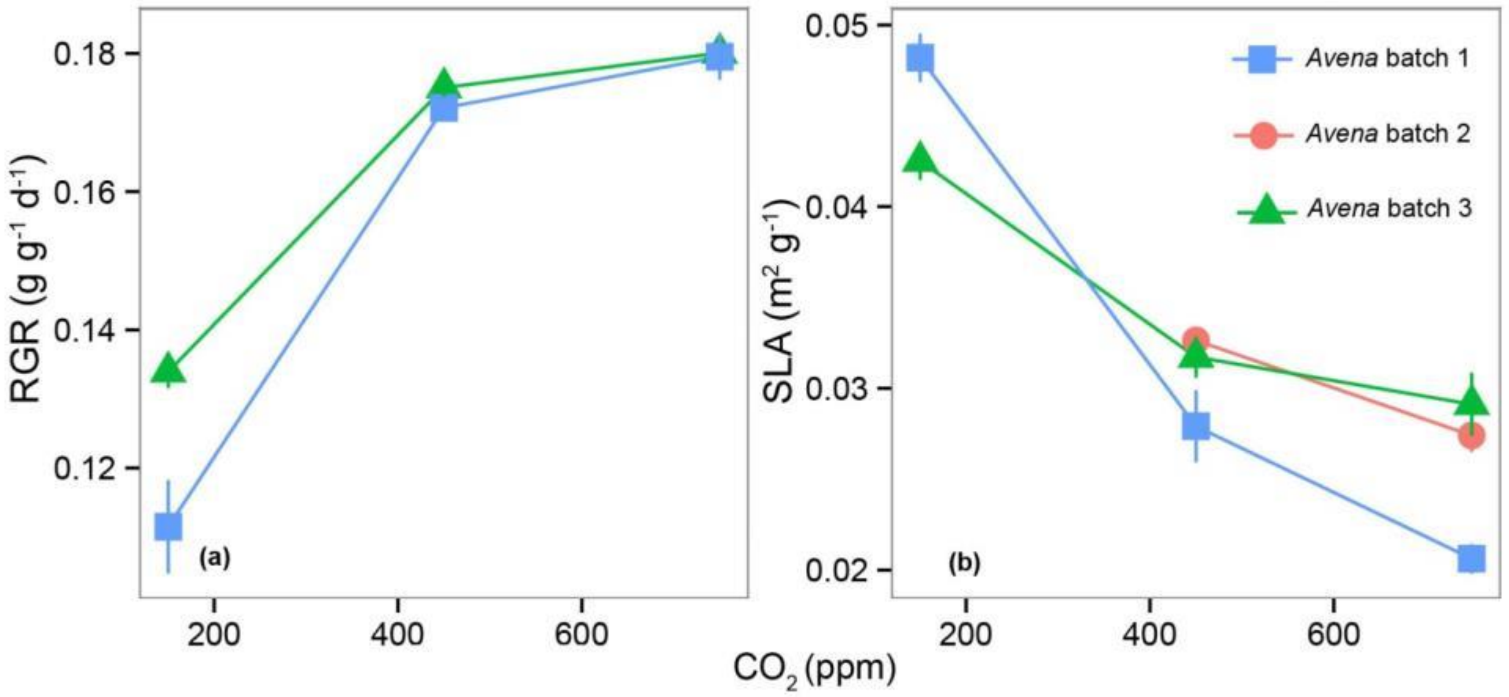

Figure S1 Response of multiple batches of *Avena sativa* to CO_2 grown at different times during the experiment. **(a):** Relative growth rate (RGR), **(b):** specific leaf area (SLA). Plants in batch 1 were for a pilot experiment, batch 2 had missing low CO_2 data, batch 3 was used for the main results

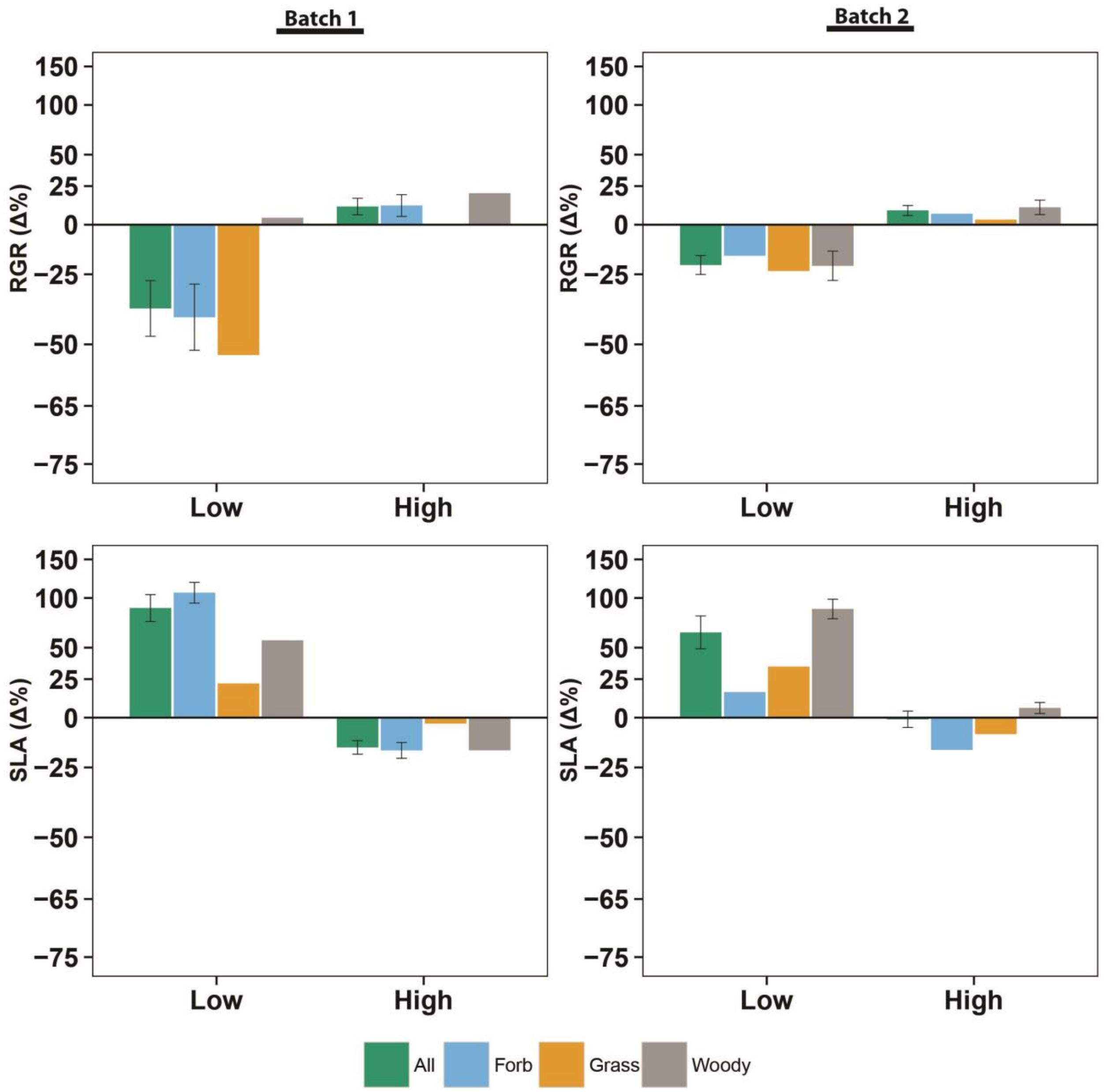

Figure S2 Comparison of RGR and SLA response to CO_2 of two batches of different species grown at different times. Relative shift in trait level at low or high CO_2 compared to ambient CO_2 for forb, grass and woody species. Bars indicate percentage shift in trait value at low CO_2 (160 ppm) and high (750 ppm) CO_2 compared to trait value at ambient (450ppm) CO_2. Axes are natural log transformed so that the size of the bars at a 50% decrease or a 100% increase is the same (reflecting a factor 2 adjustment). Green bars: all species, blue bars: forb species, orange bars: grass species, grey bars: woody species. Error bars give SE if there are multiple species per type.

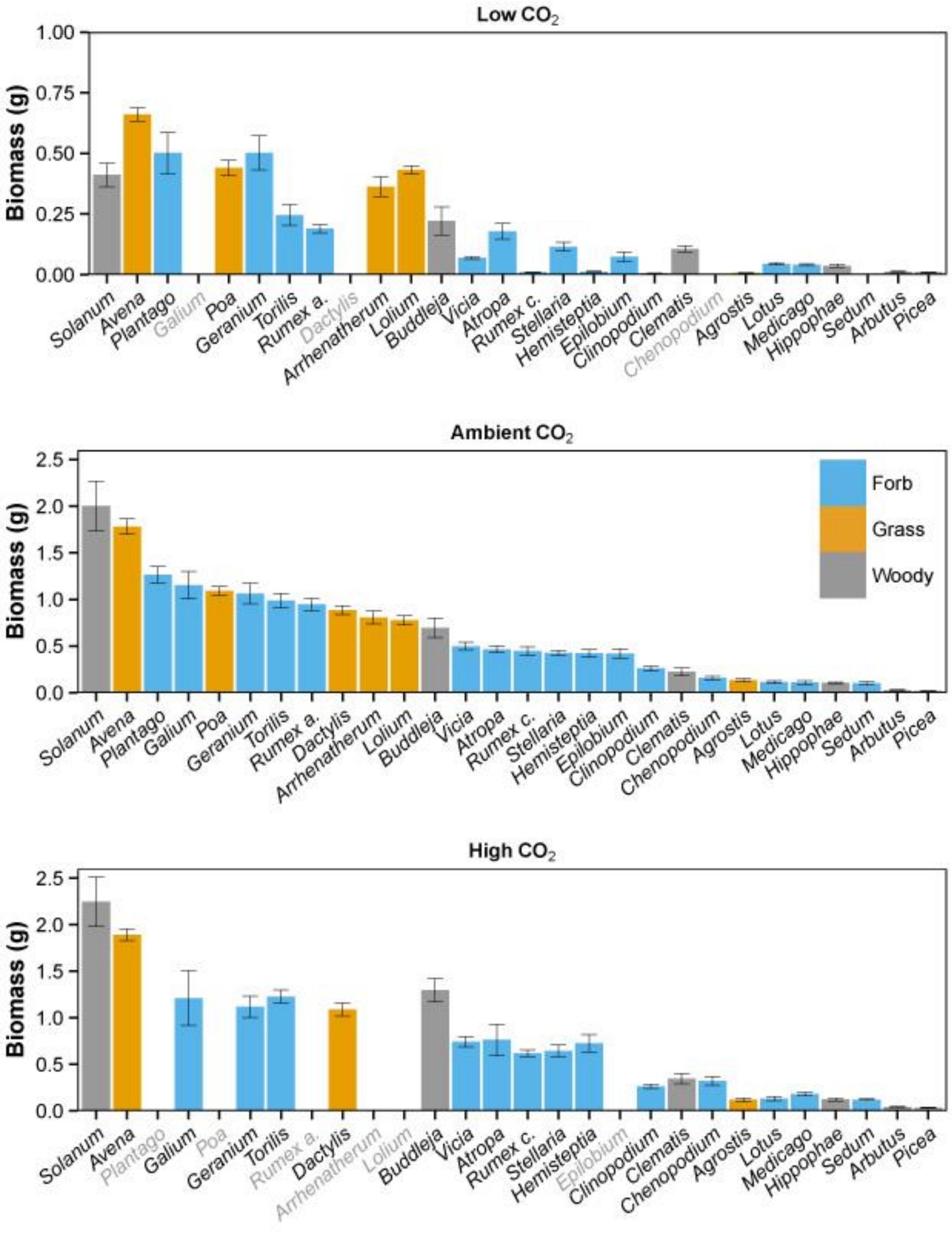

Figure S3 Plant species biomass (g) ranking at 160 ppm, 450 ppm and 750 ppm CO_2. Species are ordered by RGR at 450 ppm CO_2. Light grey species names indicate species is missing at this CO_2 treatment. Note the different axis scale at low CO_2. Orange bars: grass species, blue bars: forb species, grey bars: woody species. Error bars denote SE.

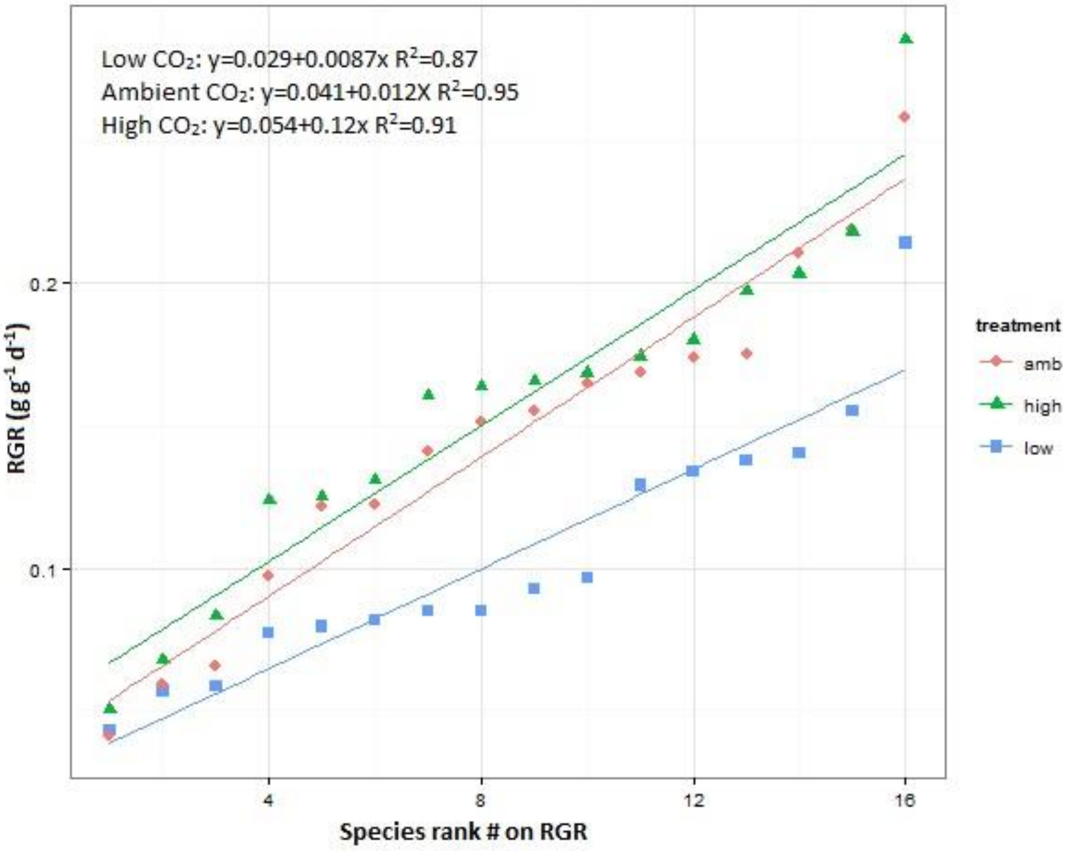

Figure S4 Plant relative growth rate rank vs Relative Growth Rate at 160 ppm, 450 ppm and 750 ppm CO_2. Species are ordered by RGR from low to high at each CO_2 level. The slope between rank and RGR is significantly lower than the slopes at ambient and high CO_2 ($p<0.05$) which shows that the difference between the fastest and slowest grower is reduced. Red: 450 ppm, Green: 750 ppm, Blue: 160 ppm

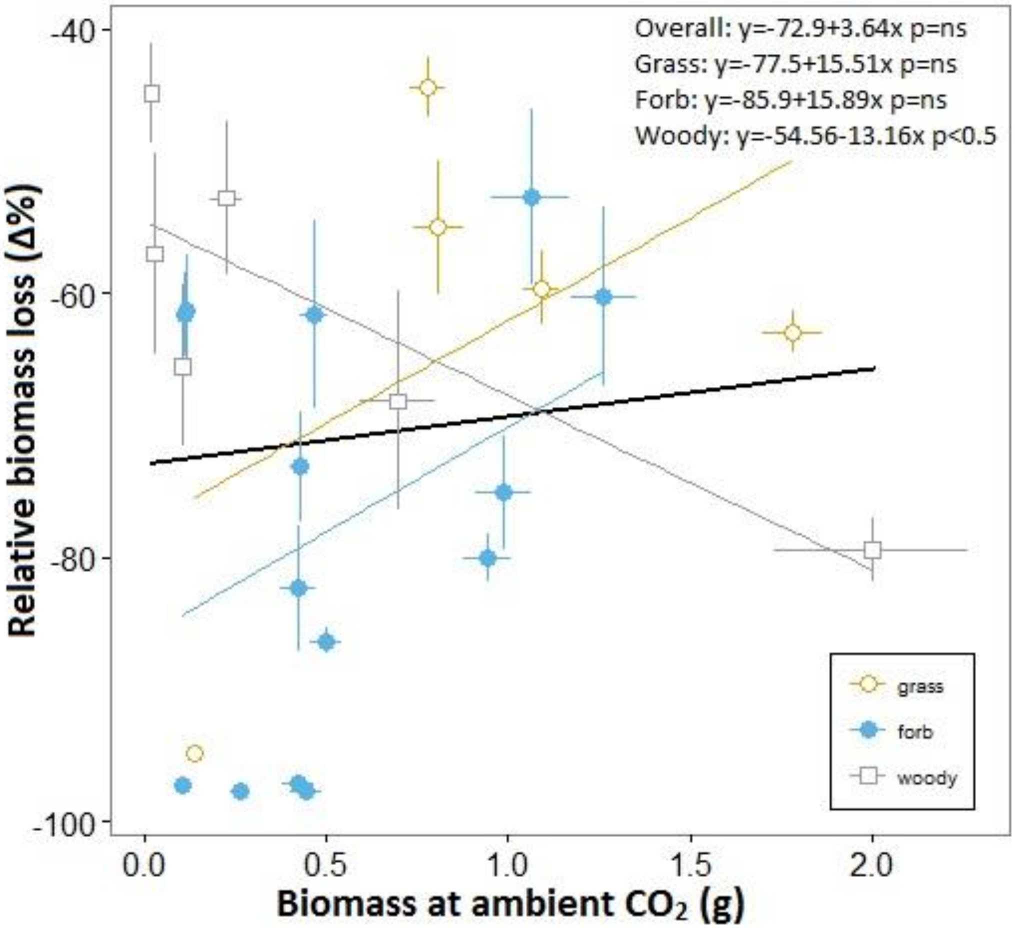

Figure S5 Relative amount of biomass lost at low (160) CO_2 as compared to ambient CO_2. Relative biomass difference (Δ%) at low CO_2 shows no relationship with biomass at ambient CO_2. Blue circles: forb species, orange open circles: grass species, grey open squares: woody species, Black line: overall regression. Error bars give SE

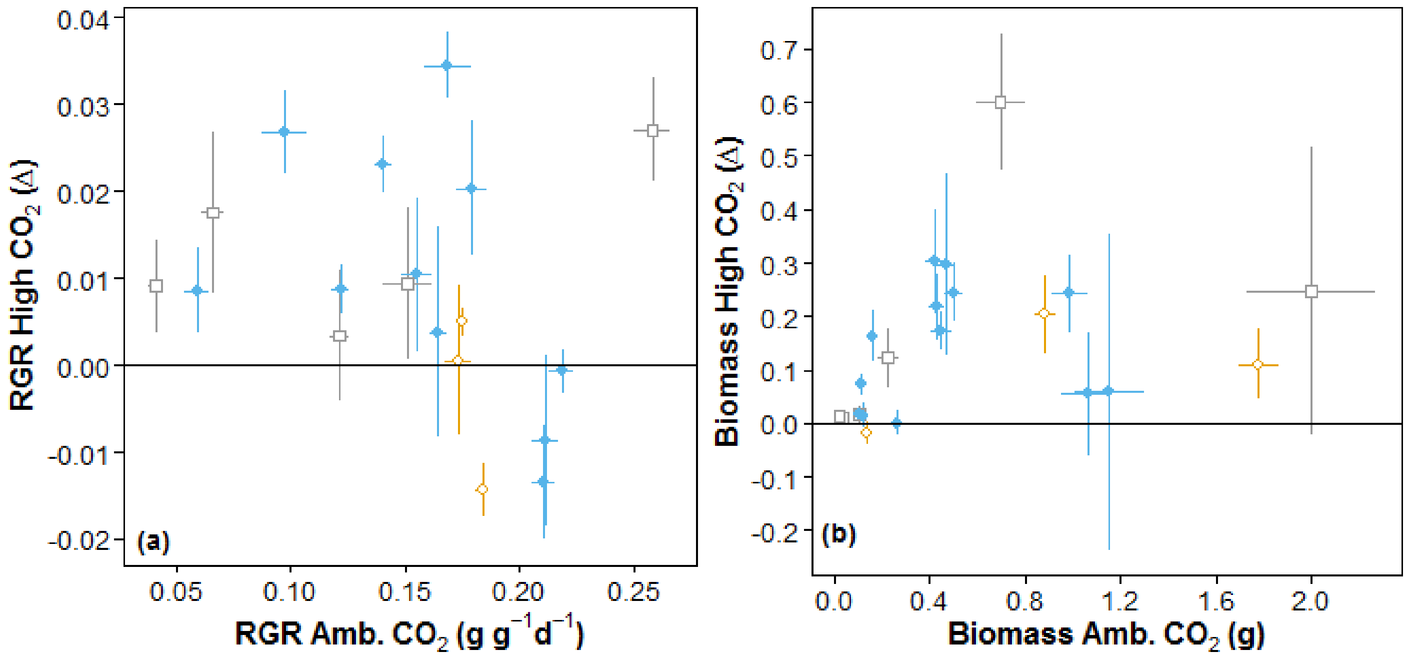

Figure S6 Difference in growth rate and plant biomass at future high (750 ppm) CO_2 compared to current ambient (450 ppm) CO_2. **a.** Relative Growth Rate difference at low CO_2 shows no relationship to growth rate at ambient CO_2. **b.** Biomass difference at low CO_2 shows no relationship with biomass at ambient CO_2. Blue circles: forb species, orange open circles: grass species, grey open squares: woody species. Error bars give SE

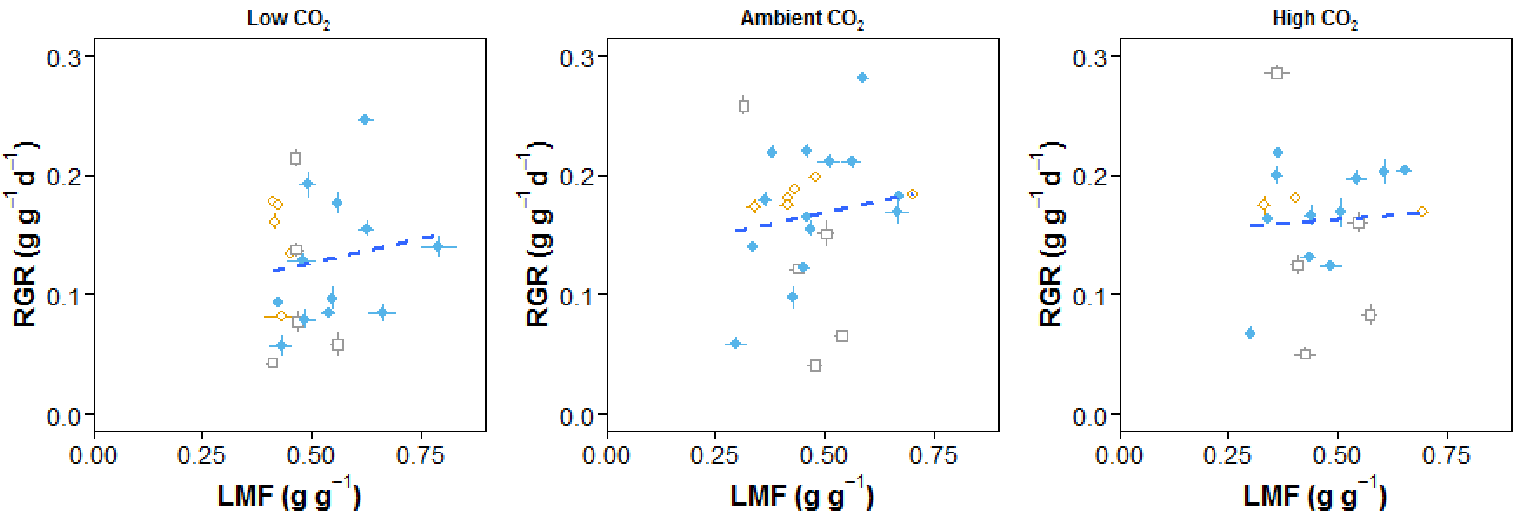

Figure S7 Relationship between leaf mass fraction (LMF) and relative growth rate (RGR) at past low (160 ppm), current ambient (450 ppm) and future high (750 ppm) CO_2. No significant relationship between LMF and RGR was found. Points indicate species mean RGR and LMF with SE; Blue circles: forb species, orange open circles: grass species, grey open squares: woody species.
